# Reclaiming Anatomy as Method: From Morphological Reasoning to Clinical Relevance

**DOI:** 10.1002/ca.70074

**Published:** 2025-12-29

**Authors:** Katia Cortese, Marco Frascio

**Affiliations:** ^1^ DIMES, Department of Experimental Medicine, Human Anatomy, MorphoLAB University of Genoa Genoa Italy; ^2^ IRCCS Ospedale Policlinico San Martino Genoa Italy; ^3^ DISC, Department of Surgical Sciences and Integrated Diagnostics, General Surgery University of Genoa Genoa Italy

**Keywords:** academic identity, anatomical education, cryo‐electron tomography, digital anatomy, molecular reductionism, morphological reasoning, spatial biology

## Abstract

In recent decades, molecular biology and omics technologies have profoundly reshaped biomedical research, with genomics, proteomics, and other high‐throughput approaches dominating scientific agendas and funding priorities. Within this molecular paradigm, however, the anatomical sciences face an epistemic and institutional tension: morphology, historically grounded in the study of form, structure, and spatial relationships, is increasingly framed as merely descriptive or obsolete. This Viewpoint moves beyond the familiar narrative of a “decline of anatomy” to argue for its strategic reinvention as a core scientific method. Anatomy is not simply a body of knowledge but a way of seeing and reasoning that remains essential for understanding biological systems. Morphological thinking—linking structure to function in situ—provides integrative insights that cannot be derived from molecular data alone. Based on historical perspectives, epistemology, and recent advances in imaging and integrative methodologies, we show how anatomy continues to drive hypothesis generation, biomedical innovation, and clinical decision‐making. Using the Italian academic system as a case study, we highlight the growing institutional disconnect between anatomical teaching and morphologically grounded research, exacerbated by metric‐driven evaluation frameworks. Finally, we propose a roadmap for embedding morphology within emerging platforms such as spatial biology, high‐resolution imaging, and AI‐assisted analysis, reclaiming anatomy as a methodological compass for navigating biological complexity and clinical translation.

## 1 Introduction

### 1.1 Molecular Reductionism Versus Morphological Thinking

#### 1.1.1 The Promise and Limits of Molecular Detail

Molecular and omics‐driven approaches have revolutionized our understanding of biology, enabling the detailed cataloguing of cellular and tissue components, including gene expression profiles, signaling networks, and molecular alterations in development and disease (Chu et al. 2024; Gutierrez Reyes et al. 2024; Misra et al. 2019). These advances are often presented as offering a “complete” understanding of biological systems. However, an exclusive focus on molecular parts risks obscuring the importance of biological organization as a whole. The question we raise here is not whether anatomy is in decline—a topic already widely discussed—but how it can be reactivated as a generative method for the 21st century. We argue that anatomy must evolve from a perceived descriptive legacy into an integrative framework that combines morphological reasoning with advanced imaging and spatially resolved molecular data (Falletta et al. 2025).

### 1.2 Morphology as Integrative Reasoning

Morphological thinking in anatomy is fundamentally integrative: it connects form to function, situates cells within their microenvironment, and interprets the architecture of tissues and organs in situ (Megas et al. 2024). Where molecular biology often isolates individual genes or proteins, morphology emphasizes context—how structures are spatially arranged, how they interact, and how they contribute to biological function. Critics of molecular reductionism have long noted that living systems cannot be fully understood by disassembling them into molecular components alone (Mazzocchi 2011; Pyrkov et al. 2024). The French philosopher and physician Georges Canguilhem cautioned against reducing biology to physics and chemistry, arguing that such reductionism “deprives biology of a proper field of study” (Canguilhem 1991). In this perspective, the phenotype—the observable form and structure of organisms—is not a trivial downstream consequence of genes, but a primary source of biological insight. Morphological observations often generate hypotheses: the identification of a novel cell type, an unexpected tissue organization, or a structural alteration in disease can raise functional questions that purely molecular analyses may overlook. Anatomists have traditionally acted as custodians of form, mapping not only what is present but why it is organized in a particular way and what this organization implies for function and development. To dismiss this work as “merely descriptive” reflects a fundamental misunderstanding of anatomical reasoning.

### 1.3 Description as Hypothesis

In anatomy, description is not a rote cataloguing of parts but a form of analytical reasoning. As the art historian Georges Didi‐Huberman has argued in his studies of medical imagery, describing the visible is itself a way of constructing knowledge: scientific observation is an interpretive act that both reveals and shapes understanding. Jean‐Martin Charcot's photographic atlases, for example, did not simply document hysteria; they defined it visually, illustrating how bodily phenomena are framed through cultural and interpretive lenses (Didi‐Huberman 2003). These perspectives highlight that anatomical knowledge is produced through an active process of seeing, comparing, and interpreting, rather than passively copied from nature. Yet in contemporary research culture, anatomical and morphological methods are often undervalued. Terms such as “descriptive anatomy” are frequently used pejoratively, implying a lack of hypothesis‐driven rigor or modern relevance—an assumption that this paper seeks to challenge.

## 2 The Persistence and Innovation of Morphological Methods

### 2.1 Classical Tools, Modern Relevance

Classical techniques, histology, transmission electron microscopy (TEM), immuno‐electron microscopy, and even hybrid approaches like correlative light and electron microscopy (CLEM) are sometimes viewed as outdated. This is simplistic and ultimately misleading (Xu et al. 2025; Zinchenko et al. 2023). Claims that sequencing or high‐throughput screens have rendered visualization unnecessary overlook a basic principle of biology: structure underpins function at every level of organization. Morphological methods therefore remain essential for validating, contextualizing, and often guiding molecular findings. The history of endothelial cell biology illustrates this point. The introduction of electron microscopy led to the identification of plasmalemmal vesicles, Weibel–Palade bodies, and the structural heterogeneity of the endothelium, discoveries that laid the foundations of modern vascular biology (Ribatti 2026). More recently, anatomical and advanced imaging approaches revealed a previously unrecognized mesothelium in the brain's subarachnoid space, reshaping our understanding of cerebrospinal fluid flow and neuroimmune interactions (Kumar et al. 2025). Similarly, ultrastructural analyses of cryobiopsies from patients with severe COVID‐19 showed that the most damaged tissues contained surprisingly little viral material, suggesting that immunopathology rather than viral load may drive late‐stage injury (Cortese et al. 2022). At the subcellular scale, recent work from our group on NSC‐34 cell‐derived 3D model uncovered distinct nuclear and mitochondrial remodeling that could not have been predicted by molecular assays alone (Arnaldi et al. 2024). Allowing us to build a structurally faithful model for neurodegeneration research. Such findings enabled the development of structurally faithful models for neurodegeneration research and exemplify how direct morphological visualization generates insights inaccessible to PCR‐based or bulk omics approaches. In parallel, in situ cryo‐electron tomography and cryoEM are expanding the frontiers of structural cell biology by capturing dynamic events in near‐native states. The identification of hemifusomes—heterotypic hemifused vesicular organelle complexes marked by a 42‐nm proteolipid nanodroplet—has even led to an ESCRT‐independent model of multivesicular body biogenesis (Tavakoli et al. 2025).

### 2.2 Morphology Becomes Data

Far from being static, morphology has evolved into a quantitative and computational discipline capable of generating high‐dimensional data that complement omics. The emerging concept of the *morphome* frames morphology as a systematic and quantifiable layer of biological information, comparable to genomics or proteomics (Son et al. 2024). As Bruno Latour famously observed, scientific images function as “immutable mobiles,” circulating across laboratories and publications while carrying epistemic authority (Latour 1987). Likewise, Susan Sontag emphasized that the ways in which bodies are visualized profoundly shape how illness is understood and narrated (Sontag 1978). Cultivating visual and morphological reasoning is therefore not ancillary but central to producing biologically meaningful and humanized interpretations of molecular data.

### 2.3 Computable Anatomy and Semantic Ontologies

The role of anatomy in data science is also being reshaped by the Model Organism Database (MOD) community (Howe et al. 2021) and by large‐scale initiatives such as the Human BioMolecular Atlas Program (HuBMAP) (Börner et al. 2025) and the Human Reference Atlas (Bueckle et al. 2025). These efforts formalize anatomical knowledge through semantic ontologies, enabling structural features to be computationally linked with gene expression, molecular pathways, and functional states. By rendering anatomical descriptions interoperable and machine‐readable, such resources demonstrate how morphology can function as a computable layer of biological information rather than a purely descriptive one. In this context, ontology‐driven anatomy reinforces the concept of the *morphome* as an integrative, hypothesis‐generating framework connecting structure, function, and molecular data across scales.

## 3 Case Study—Italy: Anatomy Taught but No Longer Practiced?

The marginalization of anatomy as a research discipline is a concern in many national contexts, but the Italian academic system offers a particularly illustrative case. Faculty positions in Italy are formally designed to integrate teaching and research, a unified role that should, in principle, sustain disciplinary continuity and methodological coherence. In practice, however, this integration is often incomplete.

At many institutions, anatomy remains a cornerstone of medical curricula, taught to large cohorts of students across multiple degree programs. At the same time, the research activities of faculty assigned to anatomy courses frequently fall outside the morphological domain, focusing instead on areas such as immunology, biochemistry, or molecular biology. Although these fields bring valuable expertise, the result can be a structural disconnect: anatomy is taught, but not actively practiced as a scientific method grounded in imaging, spatial reasoning, and structural analysis.

This divergence reflects both systemic pressures and the cumulative effects of recruitment patterns and shifting research priorities over time. Research evaluation procedures—such as Italy's national assessment system (VQR)—tend to emphasize publication volume, journal impact factors, and alignment with high‐prestige molecular domains. Several studies have criticized these frameworks for reinforcing quantity‐over‐quality incentives, disadvantaging hypothesis‐driven or exploratory research, and narrowing the scope of scientific inquiry (Abramo and D'Angelo 2016; X. Wang 2022). Within such evaluation systems, investigators working with advanced imaging, ultrastructural analysis, or integrative morphology may struggle to compete, despite producing conceptually rigorous and methodologically sophisticated work.

What emerges is a form of *disciplinary simulation*: anatomy persists as an institutional label and a teaching responsibility, while its methodological foundations as a research discipline become increasingly fragile. The risk is that anatomy is reduced to a teaching‐only field, disconnected from the investigative logic that once made it scientifically generative. Importantly, this situation is neither unique to Italy nor universal within it. Comparable tensions between teaching load, research specialization, and evaluation metrics have been reported in other academic systems (Ramirez‐Montoya et al. 2023; Wang et al. 2024). The value of the Italian case lies precisely in its capacity to foreground a broader structural issue: when research output is assessed independently of disciplinary methods, core scientific approaches can be marginalized—even within the institutional spaces nominally dedicated to them.

## 4 A Clinical Perspective: Why Surgeons Still Need Anatomical Thinking

### 4.1 Anatomy as Clinical Literacy

This disconnection is not merely an academic concern; it has direct consequences for clinical training and patient care. From a surgical perspective, the marginalization of anatomy represents a practical risk rather than a theoretical one. Surgical training relies on a deep and precise understanding of human structure, including anatomical variants, tissue planes, and three‐dimensional relationships that cannot be fully captured by molecular data or digital models alone (Agosti et al. 2023; Ayre et al. 2022; Kashtiara et al. 2024; Shah et al. 2020). Although imaging technologies have advanced dramatically, their interpretation still depends on a foundational spatial literacy cultivated through anatomical education. Innovation in surgical practice—ranging from minimally invasive and robotic techniques to image‐guided procedures—demands not less anatomy, but more: a dynamic and integrated understanding of structure in action (Dagnino and Kundrat 2024; Li et al. 2024). Preoperative planning, intraoperative navigation, and postoperative recovery all benefit from detailed anatomical maps produced by morphologically informed research. In this context, surgeons rely on anatomists not only as educators, but as partners in advancing precision medicine (Shahrezaei et al. 2024; Singh and Tubbs 2015; Streith et al. 2022).

### 4.2 Innovation and Hybrid Bodies

Digital technologies—including 3D reconstructions, virtual reality simulations, digital twins, and augmented reality in the operating room—have transformed how anatomy is accessed, taught, and navigated (Mekki et al. 2025). Yet these tools are only as effective as the user's morphological competence. Navigating a digital or virtual body requires the same spatial literacy and interpretive skills developed through traditional anatomical training (Neyem et al. 2025). Moreover, the contemporary clinical body is increasingly hybrid: technologically augmented, implanted, mediated by devices, or modified through genome editing (Kassanos and Hourdakis 2025; Musunuru et al. 2025; Patrick‐Krueger et al. 2025). From prosthetic joints and vascular stents to neuromodulation systems and robotic interfaces, the human form is no longer biologically “pure.” Understanding such semi‐technological bodies requires renewed morphological reasoning—one that integrates structure with function, matter with machine, and variation with visualization. Without this interpretive capacity, even the most advanced digital tools risk becoming superficial. Morphological thinking enables surgeons and clinicians to translate abstract models into effective interventions, grounded in the complexity of embodied, variable, and technologized human bodies (Elharram et al. 2017). In this sense, anatomy is more relevant than ever, and it is being called to evolve alongside the bodies it seeks to understand. In this sense, anatomy is not diminishing in relevance; it is being called to evolve alongside the bodies it seeks to understand.

## 5 Reclaiming Morphology: Towards Integration and Innovation

If the clinical relevance of anatomy is clear, what concrete steps are needed to reintegrate it structurally and culturally into the biomedical enterprise? The solution is neither a retreat into rigid disciplinary boundaries nor the dilution of anatomical identity within generic interdisciplinarity. Rather, morphology must be reclaimed as a foundational method through coordinated action across education, research, technology, and institutional structures.

### 5.1 Education and Training

First, training must be rethought. Graduate and postgraduate curricula should include imaging literacy and morphological interpretation as core competencies rather than peripheral or optional skills (Klement et al. 2011). (Iwanaga et al. 2021). What might be termed Morphological Sciences should integrate gross anatomy, microscopy, digital image analysis, and three‐dimensional modeling across biological and clinical domains. While digital tools and active learning strategies, including gamification, have been shown to improve student engagement and confidence in anatomy education (Berger et al. 2025), the challenge remains to translate engagement into durable spatial reasoning and genuine morphological understanding. These competencies are central to understanding form–function relationships in health and disease and to preparing future clinicians and researchers to work effectively with complex spatial data.

### 5.2 Embedding Morphology in Research

Second, morphology must be actively embedded in research practice. Anatomists should participate in experimental design, analysis, and interpretation from the earliest stages of investigation, ensuring that structural variables are considered alongside molecular ones. This is particularly critical as spatial transcriptomics, tissue atlases, and multimodal imaging platforms expand (Chelebian et al. 2025). The interpretation of spatial omics and imaging data depends fundamentally on accurate anatomical mapping and tissue contextualization (Asp et al. 2020). Within this framework, anatomists can play a crucial role in artificial intelligence– and machine learning–based imaging by providing expert ground‐truth annotations and interpretive frameworks that guide algorithm training and validation—an increasingly recognized requirement in biomedical AI (Esteva et al. 2019; Komura and Ishikawa 2018). Without such morphological supervision, computational outputs risk losing biological and spatial meaning, reinforcing the need for close collaboration between anatomists, data scientists, and experimental biologists to ensure clinically and biologically interpretable results (Topol 2019).

### 5.3 Technology and Computation

Third, technological innovation should be embraced strategically. Digital pathology, AI‐driven morphometrics, three‐dimensional reconstruction, and virtual or augmented reality are not threats to traditional methods but extensions of morphological reasoning into computational and immersive environments (Cortese and Falletta 2025; Iwanaga et al. 2023; Joseph et al. 2025). Rather than replacing the anatomist, these tools amplify the capacity to detect structural patterns, integrate multiscale data, and translate morphology into actionable knowledge.

### 5.4 Institutional Alliances and Advocacy

Finally, alliances must be strengthened. Interdisciplinary institutes focused on imaging science, spatial biology, or surgical innovation provide fertile ground for collaboration. Anatomists, radiologists, surgeons, pathologists, and data scientists can jointly advance research that is structurally informed and clinically meaningful (Patel and Smith 2023; Shah et al. 2020). Interdisciplinarity, however, should integrate—not displace—anatomical expertise. Reclaiming morphology also requires advocacy: the anatomical community must secure funding streams, journal space, and institutional recognition that support morphologically driven discovery. Without such measures, anatomy risks being reduced to a teaching‐only discipline, with its visual logic and structural reasoning progressively lost.

### 5.5 Funding and Recognition Strategies

**FIGURE 1:**
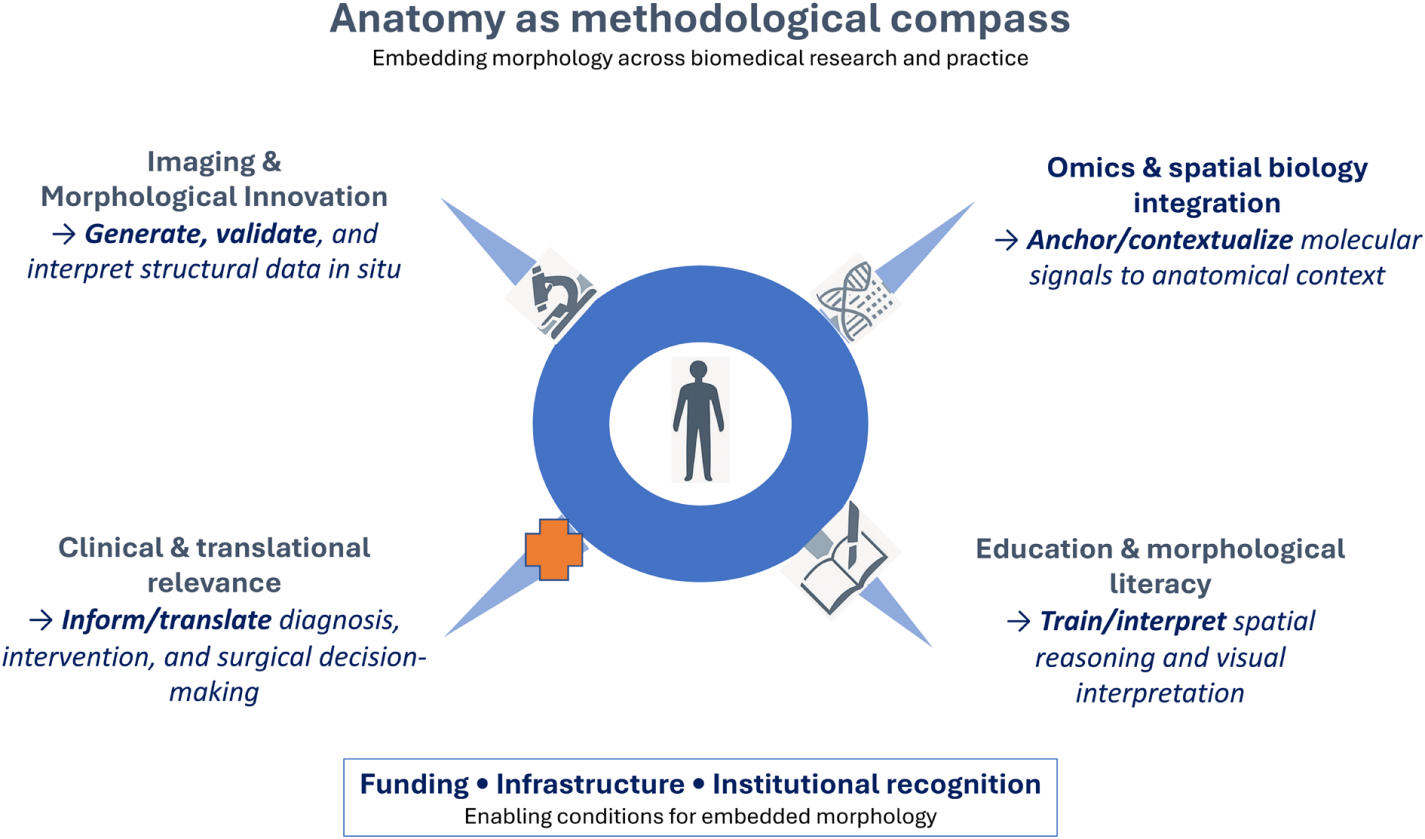
Anatomy as a methodological compass. Morphological reasoning is embedded across imaging, molecular integration, clinical practice, and education, acting as a unifying framework that anchors structure, function, and interpretation across biomedical research and training.

Securing long‐term support for morphology requires targeted and visible mechanisms. Dedicated funding streams for imaging‐driven and spatially resolved research—including shared imaging infrastructures and interdisciplinary grant calls—can provide structural stability for morphological innovation. Institutional recognition can be strengthened by embedding morphology within cross‐cutting research centers (e.g., spatial biology, surgical innovation, and digital pathology) and by creating protected journal space for morphologically driven studies. Together, these measures help ensure that anatomical research is evaluated for its conceptual and translational value, rather than marginalized by metrics that privilege molecular throughput alone. A conceptual summary of this reintegration is presented in Figure 1, which frames anatomy as a methodological compass aligning imaging innovation, molecular integration, morphological education, and clinical translation.

## 6 Conclusion: Anatomy as a Compass for Contemporary Science

If anatomy is to reclaim its place in contemporary science, the goal is not to revive past traditions but to evolve into a methodological compass for the 21st century. Advanced imaging, cryo‐electron microscopy, and correlative approaches are no longer merely technical tools; they are conceptual lenses through which biological complexity becomes legible (Pyrkov et al. 2024). Contemporary morphology integrates quantification, spatial analysis, and dynamic visualization, linking molecular signals to the architecture of cells, tissues, and organs (Pereira et al. 2016). In this sense, spatial biology, multiplex imaging, and AI‐powered morphometrics are not departures from anatomy but its natural extension, as exemplified by initiatives such as the Human Cell Atlas that integrate transcriptomic and imaging data (Rood et al. 2025; Zhang et al. 2022). Beyond basic research, anatomy plays a crucial role in the development and validation of engineered tissues, organoids, and biomaterials, where assessing microarchitecture, integration, and morphological fidelity is essential (Sean et al. 2024; Zhu et al. 2025).

This epistemic core also sustains education and clinical practice. Although digital anatomy and virtual dissection offer powerful learning tools, the tactile and visual experience of real dissection remains unmatched for developing spatial literacy and for appreciating anatomical variability through direct observation (Ghosh 2017; Krebs and Hildebrandt 2025; Sanders et al. 2025). In this way, anatomy's engagement with the visual and material body uniquely positions it to bridge biomedical science with humanistic reflection, providing not only structural knowledge but also ethical and existential insight into what it means to inhabit a human form (Arun et al. 2024; Cornwall et al. 2024). For clinicians, the implications are concrete. Mapping nerve courses and anatomical variants improves surgical precision; understanding tissue microarchitecture informs biopsy interpretation and pathology; and high‐resolution three‐dimensional models enhance preoperative planning and medical education (Grimes et al. 2026); (Andolfi et al. 2016; Darius et al. 2025). When morphology advances, clinical care improves—not as a parallel enterprise, but as a direct extension of knowing structure in context.

In short, anatomy is no longer a residue of pre‐molecular science but a way of seeing that unites structure and function, matter and meaning. Reclaiming morphology as a central method is both a scientific and a cultural task. It ensures that biomedical research remains spatially grounded, clinically relevant, and humanistically aware. If this vision is sustained, anatomy will not merely survive; it will continue to lead.

## Funding

The authors have nothing to report.

## Ethics Statement

The authors have nothing to report.

## Consent

The authors have nothing to report.

## Data Availability

This Viewpoint does not report new experimental data. All arguments and reflections are based on previously published literature, which is appropriately cited throughout the text. No new datasets were generated or analyzed for this study.
